# Ecological opportunity and upward prey-predator radiation cascades

**DOI:** 10.1038/s41598-020-67181-5

**Published:** 2020-06-26

**Authors:** Mikael Pontarp

**Affiliations:** Department of Biology, Lund University, Sölvegatan 37, 223 62 Lund, Sweden

**Keywords:** Ecological modelling, Evolutionary ecology, Coevolution, Evolutionary theory

## Abstract

A general goal in community ecology and evolutionary biology is to understand how diversity has arisen. In our attempts to reach such goals we become increasingly aware of interacting ecological and evolutionary processes shaping biodiversity. Ecological opportunity and adaptive radiations can, for example, drive diversification in competitive communities but little is known about how such processes propagate through trophic levels in adaptive radiation cascades. I use an eco-evolutionary model of trait-based ecological interactions and micro-evolutionary processes to investigate the macro-evolutionary aspects of predator diversification in such cascades. Prey diversification facilitates predator radiation through predator feeding opportunity and disruptive selection. Predator radiation, however, often disconnects from the prey radiation as the diversification progresses. Only when predators have an intermediate niche width, high predatory efficiency, and high evolutionary potential can radiation cascades be maintained over macro-evolutionary time scales. These results provide expectations for predator response to prey divergence and insight into eco-evolutionary feedbacks between trophic levels. Such expectations are crucial for future studies that aim for a better understanding of how diversity is generated and maintained in complex communities.

## Introduction

A general goal in ecology and evolutionary biology is to understand how diversity is generated and maintained and it is increasingly appreciated that ecological and evolutionary processes interact in shaping natural communities^1^. For example, much of the observed diversity has arisen through eco-evolutionary interactions and adaptive radiations, mediated by ecological opportunity and niche availability following colonization of novel environments or innovation^2–4^. We are, however, only starting to understand the mechanisms of such diversification. Especially, eco-evolutionary interactions that may underpin adaptive radiations in trophic communities remain elusive. It is largely unknown whether predator diversification occurs through the filling of niche space that is constituted by the distribution of already diversified prey or if predator diversification is driven by co-evolution such that prey and predator diversification is synchronized in, so-called, adaptive radiation cascades^5^.

General theory on diversification exists for the link between niche availability^6–8^, frequency-dependent competition for resources and speciation^9–11^. Intraspecific competition can, for example, induce disruptive selection which is crucial for adaptive radiations of competitive communities. In the context of trophic interactions, it has been shown that predation can induce disruptive selection on prey populations and thus drive evolutionary branching of prey^12–14^. Such theory provides a framework for studies on ecological opportunity and adaptive radiations in a community context^15–17^. However, despite models of adaptive radiations in trophic communities^13,14,18–21^, the role of co-evolution and radiation cascades remains elusive. Especially predator diversification in response to prey diversification needs attention in our attempts to fully understand how diversity is generated and maintained in larger natural communities^5^.

**Figure 1:**
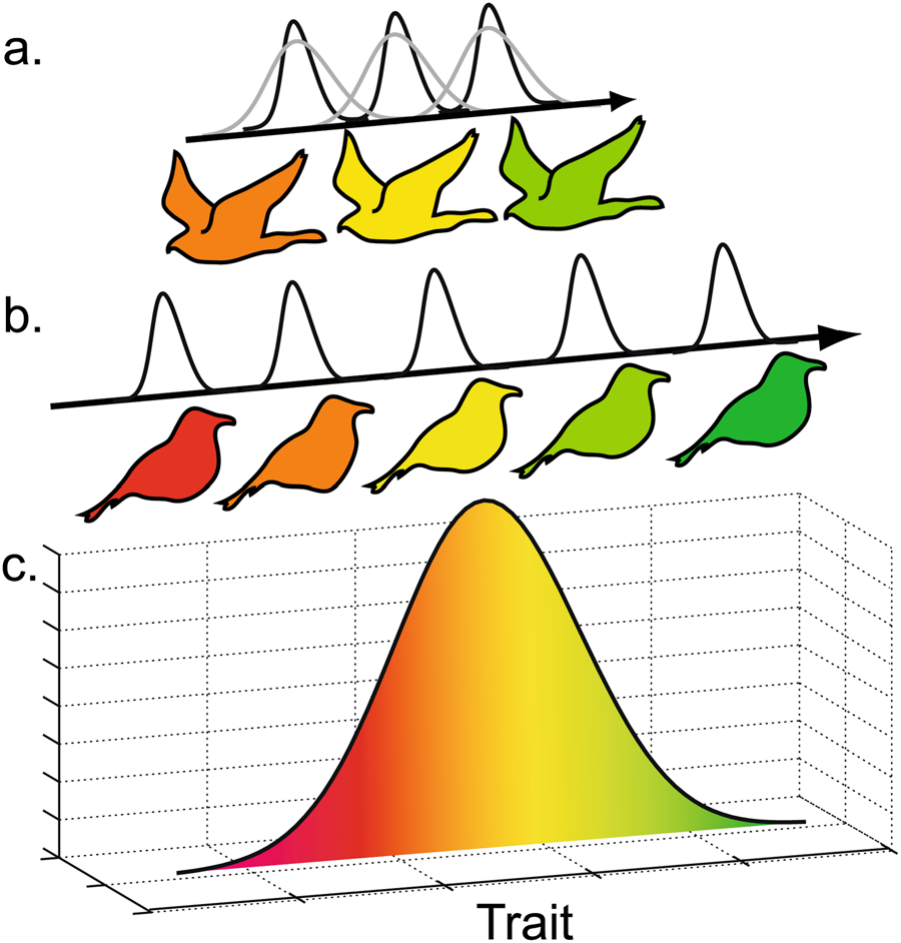
Model illustration. Top consumers (**a**) with some trait z (e.g. birds of prey with body size z) and competitive prey (**b**) with trait u (e.g. granivorous birds with beak size u) that interact in a local habitat (**c**) defined by some implicit resource distribution with peak abundance as uopt and width σK. The three trophic levels are distributed on the same trait dimension (e.g. size) here illustrated by color. Competition between species is dictated by their niche width (black and gray Gaussian kernels), and I assume that populations with similar traits interact more than less similar ones. The invasion fitness of a mutant is thus a function of its trait-matching to its resources, the traits of its competitors on the same trophic level and their niche widths. Image created in Adobe Illustrator CS6 Version 16.0.0 (64-bit).

Here, I aim to answer calls for theory associated with questions on how predators respond to prey diversification and under what circumstances upward adaptive radiation cascades can be expected. I build on a trait-based^10,20,22,23^ adaptive dynamics approach^9^ and I apply it in a predator-prey radiation context. More specifically, in line with Pontarp and Petchey^20^ I build on an ecological predator-prey model with the assumption that the diverging traits also have a direct influence on ecological interactions. This is a common assumption of trait-based eco-evolutionary models and such a trait can, for example, be the beak size of birds and their preferred resource (e.g. seeds)^17^. Body size is another such trait that has been shown to affect resource utilization and the strength of ecological interactions^24–30^. I thus follow established approaches^21,22,31,32^ by assuming that resources are distributed along some generally defined trait dimension (e.g. size) and I assume that both predator and prey populations are defined by some resource utilization trait, distributed along the same trait dimension (Fig. 1). The per capita growth (fitness) of a focal prey individual associated with a given population is a function of its resource utilization trait, its niche width, the abundance of the individual’s population, the local resource distribution and the abundance of all other populations to whom it may interact see also^15,16,33^. The fitness of a predator is a function of its traits, the traits, and abundance of its prey and the traits, and abundance of other predators to which the focal population may compete with.

I implement the ecological model in an eco-evolutionary context with connected predator-prey adaptive radiations as emergent model outputs^20,34^. Prey-resource, prey-prey and predator-prey trait matchings dictate resource utilization, competition, and trophic interactions respectively. Predators will be selected to match the trait of their prey and prey will be selected to mismatch predators while trait matching within trophic levels will be selected against due to competition for resources. A mutant (the source of phenotypic variation in the model) that occurs in trait space where many similar and abundant populations already exist or where resources are low thus tend to have low invasion fitness. Contrary, mutants in trait space where resources are available and competition is low have high invasion fitness. I utilize these properties of the model to simulate emerging adaptive radiations and I focus on the properties of predators (e.g. niche width, efficiency, and evolvability) that can lead to upward radiation cascades while at the same time considering the prey properties that may facilitate downward effects from predators on prey. More specifically, I investigate the fitness landscape of predators throughout macro-evolutionary history to assess if predators are filling niche space that is constituted of already diversified prey or if predator diversification is driven by synchronized predator-prey diversification. I also match the detailed mechanistic drivers of radiations across trophic levels to a more empirically tangible measure of congruence between prey and predator phylogenetic trees.

## Results

**Figure 2:**
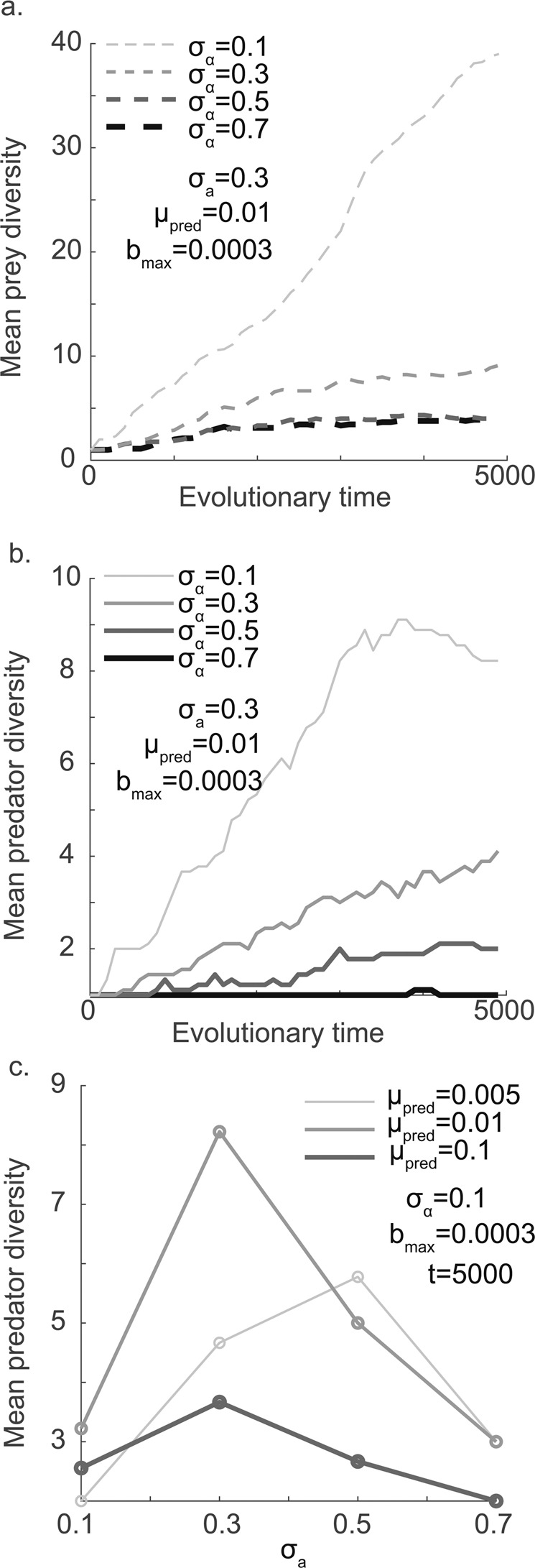
Prey and predator diversity as a function of evolutionary time and prey niche width (σ_α_) (**a,b**), and predator diversity as function of predator niche (σ_a_) width and predator mutation probability (μ_pred_) at time (*t*) equal to 5000 (**c**).

**Figure 3:**
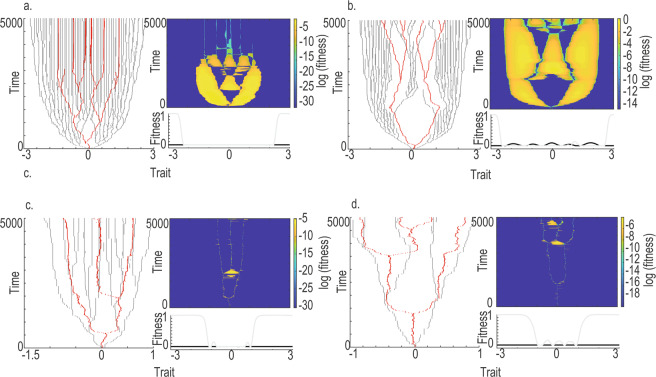
Predator (red) and prey (grey) adaptive radiations and predator fitness landscapes (heat map) in trait space, with evolutionary time ranging from 0-5000 time steps. Also, prey (grey line) and predator (black line) fitness landscape at evolutionary time t = 5000 (lower right panel). Panels in a. shows results from an example model realization with bmax = 0.0001 and μpred = 0.005. In panels b. bmax = 0.0003 and μpred = 0.005. In panels c. bmax = 0.0001 and μpred = 0.01. In panels d. bmax = 0.0003 and μpred = 0.01. Other parameters were constant at σα= 0.1 and σa=0.3.

Irrespective of other parameters (e.g. predator efficiency (b_max_), predator mutation rate (μ_pred_) or predator niche width (σ_a_)) most of the predator diversity occurs when prey niche width (σ_α_) is low (Figs. 2a,b, and S1 and S2). This is expected as a low σ_α_ facilitates the co-existence of multiple prey populations and prey branching^20^. Prey diversity is also a prerequisite for predator diversification as the distribution of prey constitutes predator niche space and there needs to exist more than one phenotypically distinct prey for disruptive selection on predators to occur (Eq. 9 in the methods section). The distribution of prey tends to be wide with many phenotypically similar prey species when σ_α_ is low (see prey radiation in Fig. 3a) which explains the positive relationship between predator diversity and σ_α_. Another striking result is a hump-shaped relationship between predator diversity and predator niche width (σ_a_) (Figs. 2c and S2). Such patterns make sense, as specialized predators are sensitive to prey that may out-evolve them. If the prey evolves to peripheral parts or outside of the predator’s niche the probability of extinction increases. In contrast, if predators are generalists the risk for prey evolving out of the predator niche width is low but multiple predator co-existence is reduced. Furthermore, high predator mutation rates (μ_pred_) tend to reduce diversity, compared to low and intermediate μ_pred_ (Fig. 2c). This is in line with Pontarp and Petchey^20^ who show that high predator mutation rates tend to disrupt diversification both in prey and predators. The results presented above provide insights into the eco-evolutionary processes of predator diversification (see for example^12–14^) but they remain silent on whether such diversification is synchronized among trophic levels due to co-evolution in adaptive radiation cascades.

**Figure 4:**
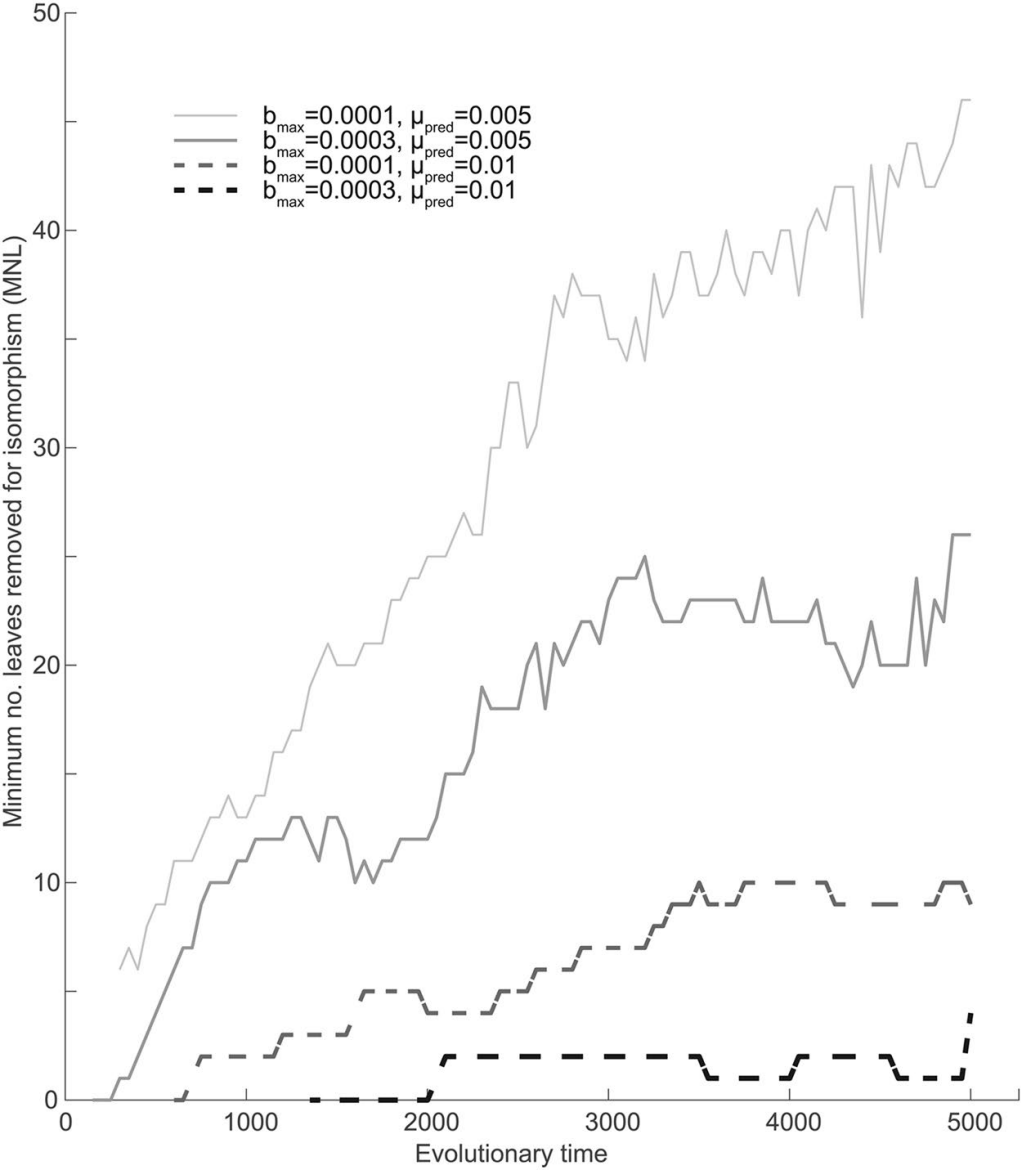
Congruence based on one model realization measured as the minimum number of leaves (in the predator and prey phylogeny) removed to reach isomorphic phylogenies, as a function of evolutionary time, for combinations of bmax and μpred combinations. Note, low MNL is related to high congruence.

Focusing on radiation cascades, results show that when predator efficiency (b_max_) and evolvability (μ_pred_) are low (Fig. 3a), radiation cascades across macroevolutionary time scales do not exist. Indeed, it follows from the model assumptions (Eqs. 2 and 9) that prey diversity is required for disruptive selection on predators to exist. All predator radiations can thus be viewed as initiated by upward radiation cascades but predators radiate largely independently, filling up niche space without co-evolution with any particular prey species. This disconnection between radiations is confirmed by large regions of high invasion fitness in the predator fitness landscapes (Fig. 3a) and low congruence among phylogenetic trees throughout evolutionary time (light gray line in Fig. 4).

Elucidating the effect of predator niche width (σ_a_), efficiency (b_max_) and evolvability (μ_pred_) on co-radiation patterns and congruence I find that a change in σ_a_ does not affect prey radiations when b_max_ and μ_pred_ are low. The disconnect in predator-prey radiations thus remains, irrespective of σ_a_. Congruence is, however, positively related to σ_a_ but this is seemingly not due to increased co-radiation but rather due to a positive relationship between predator diversity and congruence. Fewer leaves in trees also lead to fewer leaves removed to reach isomorphism. However, when predator efficiency (b_max_) is increased (Fig. 3b) predators start to affect prey radiations, disrupting prey branching and leaving gaps in prey niche space (see also^20^). As a result, the radiations reveal a more distinct pattern of predators co-evolving with prey. With this being said, the prey is still radiating outside the niche range of predators, and large parts of the prey and predator radiations are thus disconnected. This is revealed by the predator fitness landscape which shows large regions of positive invasion fitness (Fig. 3). Congruence is dramatically increased (Fig. 4) but in this case, such an increase seems to be due to the reduced prey diversity rather than actual co-radiation. When b_max_ is even larger (b_max_ = 0.0005–0.0007) predator radiations are disrupted altogether (Fig. S3a,b) as the predator pushes prey radiations away from niche space. Only when b_max_ is large in combination with a large σ_a_ can the predator seemingly co-radiate with prey but still with some prey radiating outside the range of the predators (Fig. S3c,d). These results show an interaction effect between b_max_ and σ_a_. An efficient specialist predator does not radiate as easy as an efficient generalist (results shown by the shift in diversity peak along the σ_a_ axis in Fig. S2).

Finally, the effect of an increased μ_pred_ affects prey radiation by increasing diversification rate but still when b_max_ is low such radiation remains largely disconnected from the prey radiation (Fig. 3c). Congruence increases even further (Fig. 4), plausibly due to a combination of increased co-radiation and decreased overall species richness. Ultimately, a combined increase in μ_pred_ and b_max_ provides what looks most like large co-radiations that persist throughout macro-evolutionary history (Fig. 3d). The predators radiate and they spread out in traits space such that the proportion of independently radiating prey is low. Congruence is also high, although diversity is quite low because predators reduce the radiation of prey^20^.

## Discussion

Brodersen, *et al*.^5^ list three potential predator responses to the diversification of prey. Predators may remain adapted to and specialized on one prey, predators may evolve to be a generalist, or the predator may diversify. In this paper, I expand on the mechanisms for each of these outcomes by explicitly focusing on predator properties. I focus on a combination of predator niche width, evolvability, and efficiency. Each of these properties affects the probability of predator survival and diversification and they have been shown to affect prey diversification in a downward radiation cascade^20^. In concordance with classic adaptive dynamics and radiation theory^9^, I conclude that an intermediate predator niche width facilitates predator radiation. Predator efficiency and evolvability also dictate how connected the prey and predator radiations are. Predator radiations are initiated by prey diversification but the connection between prey and predator radiations can break down soon after the first predator branching. Prey radiation is thus a prerequisite for predator diversification as it creates niche space in which predators can diversify. Whether the radiations represent radiation cascades depends on the definition of a cascade. Brodersen, *et al*.^5^ use a wide definition of cascades including seemingly disconnected cichlid radiations in Lake Victoria as an example. In this sense, my modeled radiation also falls into the category of cascades, although it can be argued that such predator radiations fall in the category of “classical” radiations as it is mainly driven by adaptation to empty niche space and competition for prey even though the niche space may be constructed by radiating prey.

In my results, radiations become more and more connected with a combined increase in predator evolutionary potential (related to mutation rate μ_pred_) and efficiency (b_max_). As predator efficiency increases, predator abundance tends to increase and predator extinction probability decreases. Increased predator population sizes also increase evolutionary potential, as do increased mutation rate. Predator evolvability thus also facilitates radiation cascades (Figs. 3 and 4). With this being said, a too efficient or a too fast-evolving predator can exclude prey from niche space, disrupting predator diversification altogether (Fig. S3). Interestingly, the diversity of the prey is also dramatically decreased in scenarios with efficient and fast-evolving predators, indicating a clear two-way process where predators affect the prey radiation and vice versa. Pontarp and Petchey^20^ go into depth about the mechanistic underpinning of how predator diversification can restrict prey radiations through decreased population sizes, decreased disruptive selection and the exclusion of prey from parts of niche space. Such a downward effect has been shown before and it questions the concept of one-way (upward or downward) radiation cascades. Does a one-way radiation cascade ever exist, and if so how can we quantify it? Such questions also open for a discussion about the possibility to determine and quantify radiation cascades from data. In a simple model with known processes, like the one analyzed here, it is relatively easy to determine if cascades occur through combined visual inspection of the radiations, fitness landscape analysis and congruence analysis. Such a wealth of information is however not available in most empirical systems which make quantification difficult. Congruence may be the only measure that is available and my analysis point to some difficulties associated with such measures. Congruence analysis is, for example, highly dependent on community diversity and null model approaches may be needed to account for such effects.

Despite the open questions about the definition and quantification of radiation cascades, the above provides sought after theory and testable expectations for when upward adaptive radiation cascades occur. For example, aquatic systems^35,36^ have been suggested to include upward radiation cascades and data are thus available for such tests. With this being said, results should be viewed in the context of the model design and specific assumptions. Although explicitly designed to model adaptive radiations in predator-prey systems the model is quite general and simplistic as it models asexual organisms, linear functional responses, and I assume a constant environment and resource availability. The model also assumes a one-dimensional trait space and it includes explicit assumptions about competition and predation through trait matching. It is thus important to note that the results presented here are relevant for when a prey trait (e.g. size or some other ecological trait) dominates and drives the response in predator traits or vice versa. The signal of radiation cascades may be less prevalent when multiple correlated functional traits^37,38^ affect eco-evolutionary dynamics within and among trophic levels. As noted in the introduction it may also seem unrealistic to compare communities that contain species with non-evolving narrow or broad niche widths, respectively. It has been shown that niche filling can occur through either evolved generalization or diversification and the outcome depends on resource diversity and resource-acquisition trade-off^39,40^. Given the scope of this paper where I explicitly focus on diversification, I exclude the possibility of generalization on the expense of diversification. I model wide resource diversity, I assume non-evolving niche widths, I do not model an acquisition trade-off, and I set parameters such that diversification can occur. The expectations about radiation cascades presented here are thus most relevant in the context of wide resource diversity and high cost of obtaining a generalist niche as this is a prerequisite for diversification^39^.

Despite the assumptions and simplifications listed above, the results provide an understanding of the fundamental link between eco-evolutionary processes and diversification and it reveals several of the mechanisms that are operating in diversifying complex communities. Such a detailed investigation is virtually impossible in experimental or field studies. By excluding some of the complications listed above the model reveals several interesting points associated with predator properties that may promote radiation cascades, the likely two-way process between co-radiating predator-prey radiations, and issues related to the quantification or causality of such cascades from data. This said I am sure this will not be the last words written about the complex and intriguing topic of diversification in complex communities.

## Methods

### Ecological model

Following Pontarp and Petchey^20^ I base my trait-based model of adaptive predator-prey radiations on the assumption that the diverging traits (e.g. body size) also have a direct influence on ecological interactions. I follow established approaches^21,22,24,32,33^ by modeling resources along some generally defined trait dimension and I assume that both predator and prey populations are defined by some resource utilization trait, distributed along the same trait dimension (Fig. 1). The per capita growth (fitness) of a focal prey individual associated with a given population is thus a function of its resource utilization trait, the abundance of the individual’s population, the local resource distribution and the abundance of all other populations to whom it may interact see also^15,16,33^. The fitness of a predator is a function of its trait, the traits, and abundance of its prey and the traits, and abundance of other predators to which the focal population may compete with. Mathematically the above formulate as:

1 $$\frac{d{N}_{i}}{{N}_{i}dt}=r+\mathop{\sum }\limits_{j=1}^{n}\frac{-r{\alpha }_{ij}({u}_{i},{u}_{j}){N}_{j}}{{K}_{i}({u}_{i},{u}_{opt})}-\mathop{\sum }\limits_{k=1}^{p}{a}_{ik}({u}_{i},{z}_{k}){P}_{k}$$

2 $$\frac{d{P}_{k}}{{P}_{k}dt}=-d+c\mathop{\sum }\limits_{i=1}^{n}{a}_{ik}({u}_{i},{z}_{k}){N}_{i}$$

where *N*_*i*_ and *P*_*k*_ denote prey and predator population size respectively ultimately modeling the ecological dynamics, in per capita form, of *n* prey populations and *p* predator populations for *i* =1 to *n*, *k* =1 to *p*. The model is essentially built on the generalized Lotka–Volterra (GLV) model^41^ but extended to include trait-based interactions as carrying capacity, the prey interactions and predator-prey interactions are formulated as trait dependent functions:

3 $${K}_{i}({u}_{i},{u}_{opt})={K}_{0}{e}^{-\frac{{({u}_{opt}-{u}_{i})}^{2}}{2{\sigma }_{K}^{2}}}$$

4 $${\alpha }_{ij}({u}_{i},{u}_{j})={e}^{-\frac{{({u}_{i}-{u}_{j})}^{2}}{2{\sigma }_{\alpha }^{2}}}$$

and

5 $${a}_{ik}({u}_{i},{z}_{k})={b}_{max}{e}^{-\frac{{({u}_{i}-{z}_{k})}^{2}}{2{\sigma }_{a}^{2}}}$$

Here, as in Pontarp and Petchey^20^, *K*_*i*_(*u*_*i*_, *u*_*opt*_) represents the carrying capacity for a monomorphic population of prey individuals with trait value *u*_*i*_ in a habitat characterized by a resource distribution with its peak resource availability at the point *u*_*opt*_. The parameter *K*_0_ denotes the maximal carrying capacity (at *u* = *u*_*opt*_) and it follows from Eq. 3 that the resource availability declines symmetrically as *u* deviates from *u*_*opt*_ according to the width of the resource distribution (*σ*_*K*_). The interaction coefficient, *α*_*ij*_(*u*_*i*_*,u*_*j*_), is a function that models the ecological interaction between the focal prey population (defined by its trait *u*_*i*_) and its competitors (defined by their traits *u*_*j*_). The competition coefficients is standardized such that *α*_*ii*_ =1 and 0 <*α*_*ij*_ < 1 (*u*_*i*_≠*u*_*j*_). The parameter *σ*_α_ determines the degree of competition between individuals given certain utilization traits and can thus be viewed as the niche width of the prey. Similarly, *a*_*ik*_(*u*_*i*_,*z*_*k*_) models the interaction between a focal predator population *k* with trait value *z* and a prey population *i* with trait value *u*. The parameter *b*_*max*_ denotes the maximum attack rate obtained when *u*_*i*_=*z*_*k*_ and this rate then falls of symmetrically as *u*_*i*_ deviates from *z*_*k*_ according to a Gaussian function with variance *σ*_*a*._ Similar to the *σ*_α_ parameter, *σ*_*a*_ can be viewed as the niche width of the predator.

### Eco-evolutionary simulation implementation

I implement the model in an eco-evolutionary context^20,34^. The prey-resource, prey-prey and predator-prey trait matchings (Eqs. 3–5) dictate resource utilization, competition, and trophic interactions respectively. Trait matching will be selected for between trophic levels while trait matching within trophic levels will be selected against due to competition for resources. A mutant that occurs in trait space where many similar and abundant populations already exist or where resources are low thus tend to have low invasion fitness. Contrary, mutants in trait space where resources are available and competition is low have high invasion fitness. I utilize these properties of the model to simulate adaptive radiations through alternating phases of determining equilibrium population abundances and the introduction of mutants as a source of phenotypic variation^34,42^.

I start the simulations by setting up the resource distribution with parameters u_opt_ = 0, σ_K_ = 1 and *K*_0_=10000. I implement the assumption of ecological opportunity by seeding the system with two monomorphic and perfectly matching populations (one prey and one predator) with trait values *u = z* = *u*_*opt*_, and I compute the equilibrium population sizes by integrating over Eqs. 1 and 2 until equilibrium or a steady state is reached (integrating from time 0 to 1000 is proven sufficient^34^). I then allow one individual of a selected population to mutate and change its traits to *u’* or *z’*, depending on what population was picked. More specifically, a single mutant population is drawn at random according to the product of the population sizes and mutation probability (*µ*_*prey*_ = 0.01 and *µ*_*pred*_ = 0.005 – 0.1). Note that I am assuming a constant *µ*_*prey*_ throughout this study while evolvability is one of the predator properties that I aim to evaluate in the context of radiation cascades, hence the range of *µ*_*pred*_ analyzed during different model realizations. Mutation size for both predators and prey are assumed to be a random trait value drawn from a normal distribution with mean equal to the trait value of the mutating population and a variance *σ*_*mut*_ = 0.02.

Following the adaptive dynamics framework^9,43^ I compute trait dependent invasion fitness for an arbitrary prey and predator mutant with trait value *u’* or *z’* according to:

8 $${G}_{prey}(u{\prime} ,\,{\bf{u}},\,{\boldsymbol{z}},\,{\bf{N}},\,{\bf{P}})=r+\mathop{\sum }\limits_{j=1}^{n}\frac{-r\alpha (u{\prime} ,{u}_{j}){N}_{j}}{K(u{\prime} ,{u}_{opt})}-\mathop{\sum }\limits_{k=1}^{p}a(u{\prime} ,{z}_{k}){P}_{k}$$

9 $${G}_{pred}(z{\prime} ,\,{\bf{u}},\,{\bf{N}})=-d+c\mathop{\sum }\limits_{i=1}^{n}a({u}_{i},{\rm{z}}{\prime} ){N}_{i}$$

where *u’* denotes the trait value of the mutant prey and *z’* denotes the trait value of a mutant predator. The vectors **u**, **z**, **N**, and **P** are defined as above containing the resident community trait distributions and abundances. If the mutant has positive invasion fitness, I continue the analysis with a mutual invasibility test, evaluating if a single individual with the mother trait can invade a population with the mutant trait at equilibrium population size. If the mutant invasion fitness is positive but mutual invasibility does not exist, the mutant will replace the resident. However, if mutual invasibility does exist, the resident and the mutant can co-exist. After the mutant is either introduced alongside the resident or is allowed to replace the resident, I recalculate the equilibrium, remove populations that may have gone extinct due to the introduction of the mutant population. Finally, before iterating into the next cycle of computation that constitutes an evolutionary time step I assign each population to a species id using a trait-based speciation definition where species are defined as populations having common descent and a continuous distribution of traits (no gaps in the trait distribution> 3* σ_µ_)^15,16^. If a gap> 3* σ_µ_ is detected in the trait distribution within an existing species, a speciation event is registered (i.e. one species branching into two). Thereafter I progress into the next evolutionary step, repeating the whole procedure for 5000 iterations.

### Model analysis

It is known from previous studies with similar models that diversity is contingent on niche width^9,10,13,20^. I build on such insights and investigate prey and predator diversity as a function of evolutionary time and prey niche width (σ_α_) ranging from 0.1–0.7 with increments of 0.2. I also analyze predator diversity as a function of predator niche width (σ_a_) ranging from 0.1–0.7 with increments of 0.2, predator efficiency (b_max_) ranging from 0.0001–0.0007 with increments of 0.0002, and predator mutation probability (μ_pred_) of 0.005, 0.01 and 0.1. Such analyses provide a general understanding of the diversification process, which I use as a basis for the investigation of radiation cascades. I initiate simulations with constant resource distribution, constant mutation model for prey and constant prey niche widths (*σ*_*α*_ = 0.1). This initiation ensures diversification of prey and I use it as a basis for my investigations of predator properties in the context of upward adaptive radiation cascades. The parameter space presented above is relevant for the diversification of predators as well as downward radiation cascades^20^. To quantify radiation cascades I analyze the fitness landscape (Eq. 9) and congruence between prey and predator phylogenetic trees measured as the maximum number of leaves removed to reach isomorphism among trees. Phylogenetic trees were constructed using data on the distance to the nearest common ancestor between the species and the MATLAB function *seqlinkage*.

Other constant model parameters for the simulations were *r* = 1, *d* = 0.2 and c = 0.3. These, as well as the parameters already presented above, produce diverse enough communities to analyze adaptive radiations within reasonable computational time. Given the stochastic components in the implementation of the deterministic ecological model as an eco-evolutionary simulation model, some variation between model realizations can occur. Each parameter combination throughout parameter space was thus replicated 10 times, the variation in predator and prey diversification was quantified (Fig. S4) and a robustness check across replicates was done on congruence (Fig. S5).

## Supplementary information

Supplementary Information.

## References

[1] Ellner SP, Geber MA, Hairston NG (2011). Does rapid evolution matter? Measuring the rate of contemporary evolution and its impacts on ecological dynamics. Ecol Lett.

[2] Yoder JB (2010). Ecological opportunity and the origin of adaptive radiations. J Evolution Biol.

[3] Losos JB (2010). Adaptive Radiation, Ecological Opportunity, and Evolutionary Determinism. Am Nat.

[4] Meyer JR, Kassen R (2007). The effects of competition and predation on diversification in a model adaptive radiation. Nature.

[5] Brodersen J, Post DM, Seehausen O (2018). Upward Adaptive Radiation Cascades: Predator Diversification Induced by Prey Diversification. Trends Ecol Evol.

[6] Schluter, D. *The ecology of adative radiations*. (Oxford university press, 2000).

[7] Grant, P. R. *Evolution on islands*. (Oxford University Press, 1998).

[8] MacArthur, H. R. & Wilson, E. O. *The theory of island biogeography*. (Princeton university press, 1968).

[9] Geritz SAH, Kisdi E, Meszena G, Metz JAJ (1998). Evolutionarily singular strategies and the adaptive growth and branching of the evolutionary tree. Evol Ecol.

[10] Dieckmann U, Doebeli M (1999). On the origin of species by sympatric speciation. Nature.

[11] Brännström Å, Johansson J, von Festenberg N (2013). The hitchhiker’s guide to adaptive dynamics. Games.

[12] Brown JS, Vincent TL (1992). Organization of Predator-Prey Communities as an Evolutionary Game. Evolution.

[13] Ripa J, Storlind L, Lundberg P, Brown JS (2009). Niche co-evolution in consumer-resource communities. Evol Ecol Res.

[14] Ito H, Shimada M, Ikegami T (2009). Coevolutionary dynamics of adaptive radiation for food-web development. Popul Ecol.

[15] Pontarp M, Ripa J, Lundberg P (2012). On the origin of phylogenetic structure in competitive metacommunities. Evol Ecol Res.

[16] Pontarp M, Ripa J, Lundberg P (2015). The biogeography of adaptive radiations and the geographic overlap of sister species. Am Nat.

[17] Pontarp M, Wiens JJ (2017). The origin of species richness patterns along environmental gradients: uniting explanations based on time, diversification rate and carrying capacity. J Biogeogr.

[18] Brännström A, Loeuille N, Loreau M, Dieckmann U (2011). Emergence and maintenance of biodiversity in an evolutionary food-web model. Theor Ecol-Neth.

[19] Sauterey B, Ward B, Rault J, Bowler C, Claessen D (2017). The Implications of Eco-Evolutionary Processes for the Emergence of Marine Plankton Community Biogeography. Am Nat.

[20] Pontarp, M. & Petchey, O. L. Ecological opportunity and predator–prey interactions: linking eco-evolutionary processes and diversification in adaptive radiations. *Proceedings of the Royal Society B: Biological Sciences* **285**, (2018). 10.1098/rspb.2017.2550 PMC5879621 29514970

[21] Loeuille N, Loreau M (2005). Evolutionary emergence of size-structured food webs. P Natl Acad Sci USA.

[22] Doebeli M, Dieckmann U (2003). Speciation along environmental gradients. Nature.

[23] Heinz SK, Mazzucco R, Dieckmann U (2009). Speciation and the evolution of dispersal along environmental gradients. Evol Ecol.

[24] Brose U, Williams RJ, Martinez ND (2006). Allometric scaling enhances stability in complex food webs. Ecol Lett.

[25] Leyequien E, de Boer WF, Cleef A (2007). Influence of body size on coexistence of bird species. Ecol. Res..

[26] Yvon-Durocher G (2011). Across ecosystem comparisons of size structure: methods, approaches and prospects. Oikos.

[27] Rudolf VHW (2012). Seasonal shifts in predator body size diversity and trophic interactions in size-structured predator-prey systems. J Anim Ecol.

[28] DeLong JP, Vasseur DA (2012). A dynamic explanation of size-density scaling in carnivores. Ecology.

[29] DeLong JP, Vasseur DA (2012). Size-density scaling in protists and the links between consumer-resource interaction parameters. J Anim Ecol.

[30] Violle C (2007). Let the concept of trait be functional!. Oikos.

[31] Christiansen FB, Loeschcke V (1980). Evolution and intraspecific exploitative competition I. One-locus theory for small additive gene effects. Theor Popul Biol.

[32] Brown JS, Vincent TL (1987). A Theory for the Evolutionary Game. Theor Popul Biol.

[33] Pontarp, M. & Petchey, O. L. Community trait overdispersion due to trophic interactions: concerns for assembly process inference. *P Roy Soc B-Biol Sci* **283**, (2016). 10.1098/rspb.2016.1729 PMC5069517 27733548

[34] Pontarp M, Brännström A, Petchey OL (2019). Inferring community assembly processes from macroscopic patterns using dynamic eco-evolutionary models and Approximate Bayesian Computation (ABC). Methods Ecol Evol.

[35] Muir AM, Hansen MJ, Bronte CR, Krueger CC (2016). If Arctic charr Salvelinus alpinus is “the most diverse vertebrate’, what is the lake charr Salvelinus namaycush?. Fish Fish.

[36] Brodersen, J., Howeth, J. G. & Post, D. M. Emergence of a novel prey life history promotes contemporary sympatric diversification in a top predator. *Nat Commun* **6**, (2015). 10.1038/ncomms9115 26365323

[37] Petchey OL, Gaston KJ (2006). Functional diversity: back to basics and looking forward. Ecol Lett.

[38] Pennekamp F (2018). Biodiversity increases and decreases ecosystem stability. Nature.

[39] Sjödin, H., Ripa, J. & Lundberg, P. Principles of niche expansion. *P Roy Soc B-Biol Sci* **285**, (2018). 10.1098/rspb.2018.2603 PMC6304067 30963885

[40] Ackermann M, Doebeli M (2004). Evolution of niche width and adaptive diversification. Evolution.

[41] Case, T. J. *An illustrated guide to theoretical ecology*. (Oxford University Press, Inc., 2000).

[42] Ito HC, Dieckmann U (2007). A new mechanism for recurrent adaptive Radiations. Am Nat.

[43] Metz JAJ, Nisbet RM, Geritz SAH (1992). How should we define fitness for general ecolgical scenarios. Trends Ecol Evol.

