# Supplementary material for: Ecological opportunity and upward prey-predator radiation cascades
Source: Sci Rep. 2020 Jun 26;10:10484. doi: 10.1038/s41598-020-67181-5 (PMC7320021; doi:10.1038/s41598-020-67181-5)
Supplement: Supplementary file 1 — Supplementary Information. [file 41598_2020_67181_MOESM1_ESM.pdf]

Ecological opportunity and upward prey-predator radiation cascades

Mikael Pontarp

Department of Biology, Lund University Biology Building, Sölvegatan 35, 223 62 Lund, Sweden

Supplementary Information

Figure S1

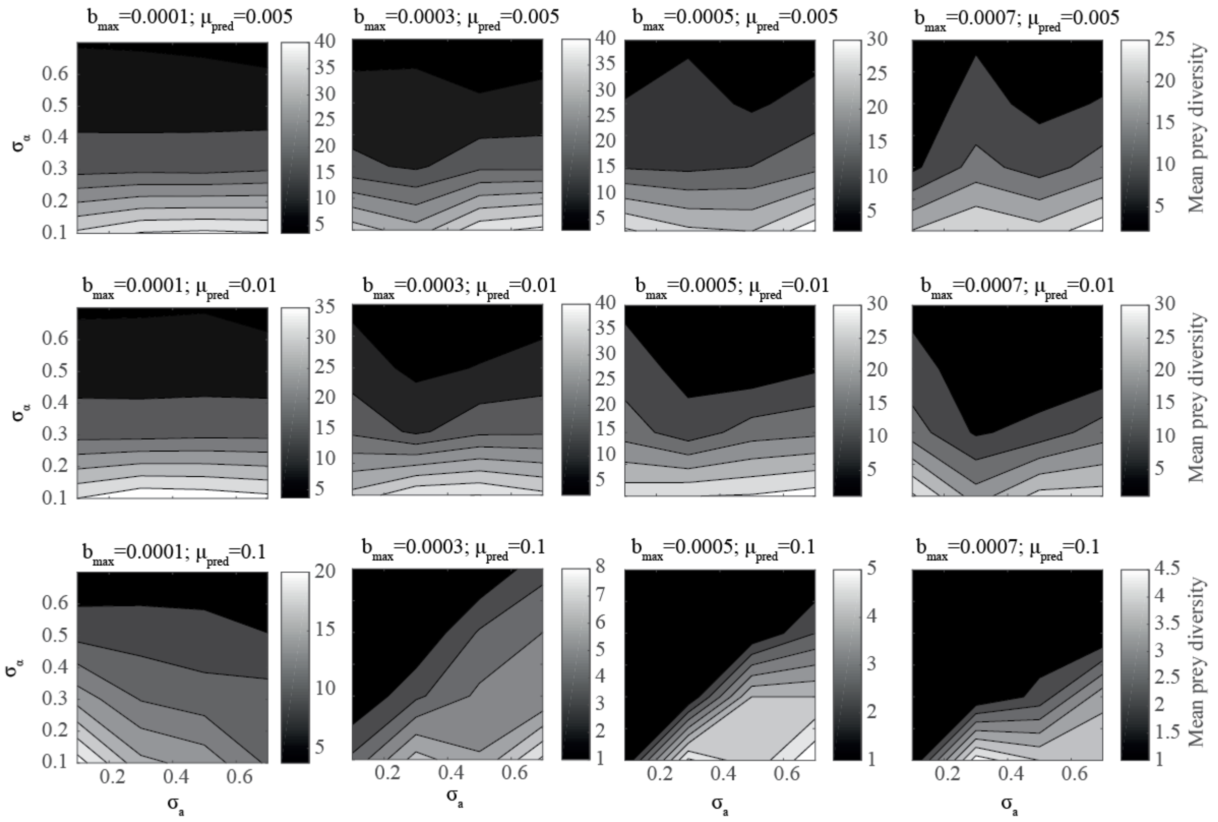

Figure S1. Mean prey diversity computed on 10 replicates throughout parameter space. Mean prey diversity was computed at evolutionary time step 5000. Constant model parameters not assigned in the figure above were r = 1, d = 0.2 and c = 0.3.

Figure S2

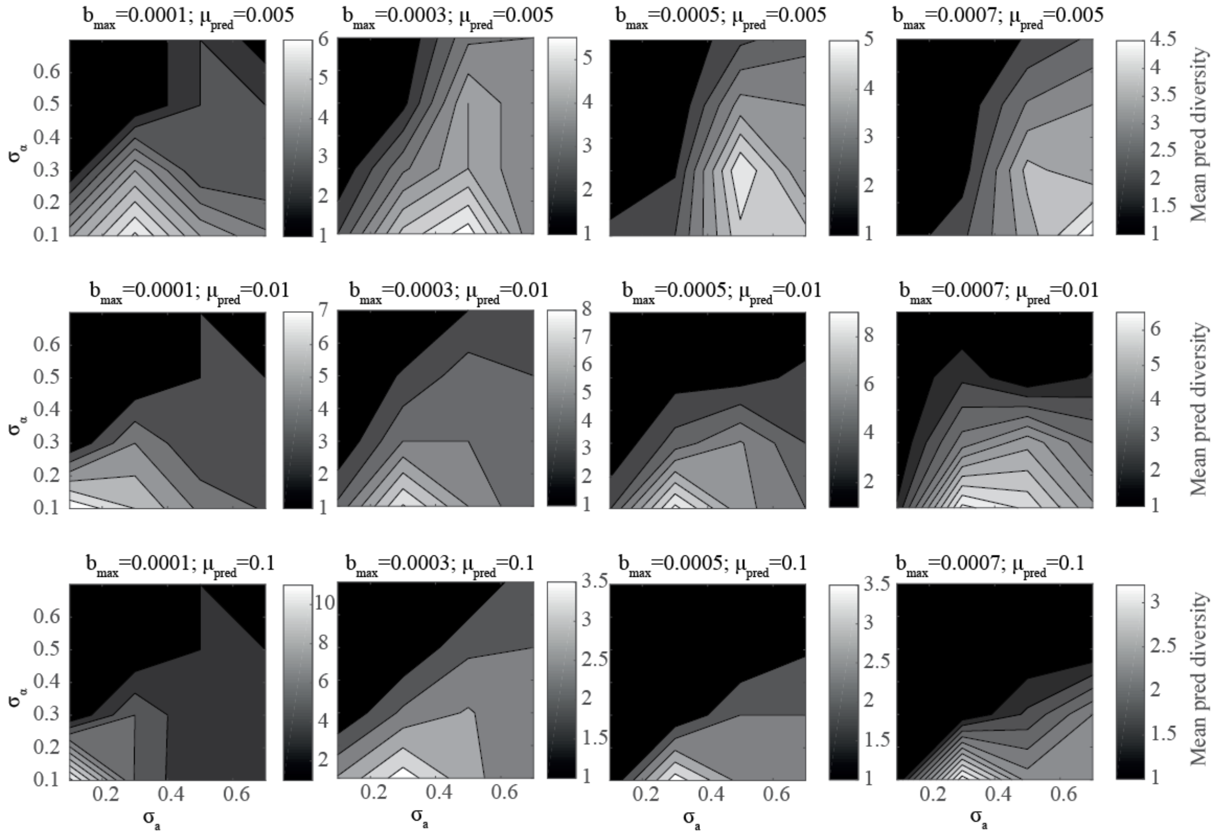

Figure S2. Mean predator diversity computed on 10 replicates throughout parameter space. Mean predator diversity was computed at evolutionary time step 5000. Constant model parameters not assigned in the figure above were r = 1, d = 0.2 and c = 0.3.

Figure S3

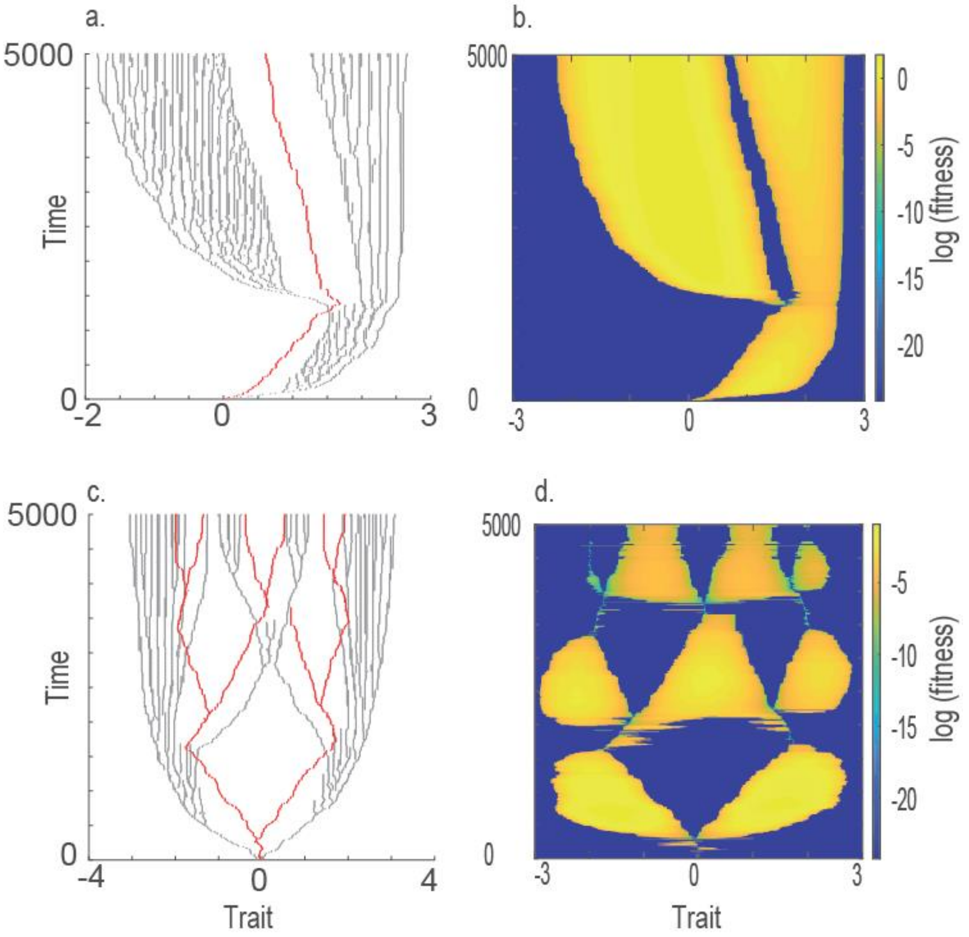

Figure S3. Predator (red) prey (grey) adaptive radiations and predator fitness landscapes (heat map) in trait space, with evolutionary time ranging from 0-5000 time steps. All panels show results from an example model realization with b_{max} = 0.0007, μ_{pred} = 0.005 and σ_{α}= 0.1. Panels a. and b. show results where σ_a=0.3 while panels c. and d. show results for σ_a=0.7.

Figure S4

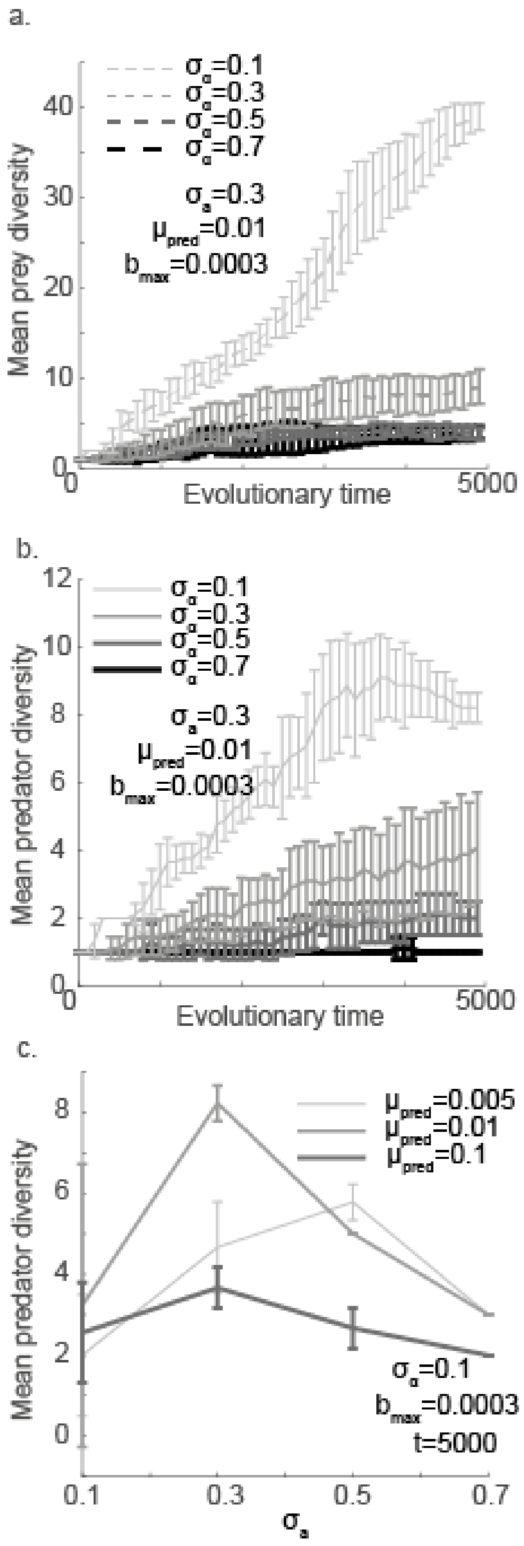

Figure S5. Prey and predator diversity as a function of evolutionary time and prey niche width (σ_α) (a, b), and predator diversity as function of predator niche (σ_a) width and predator mutation probability (μ_{pred}) at time (*t*) equal to 5000 (c). Error bars represent the standard deviation for each mean value.

Figure S5

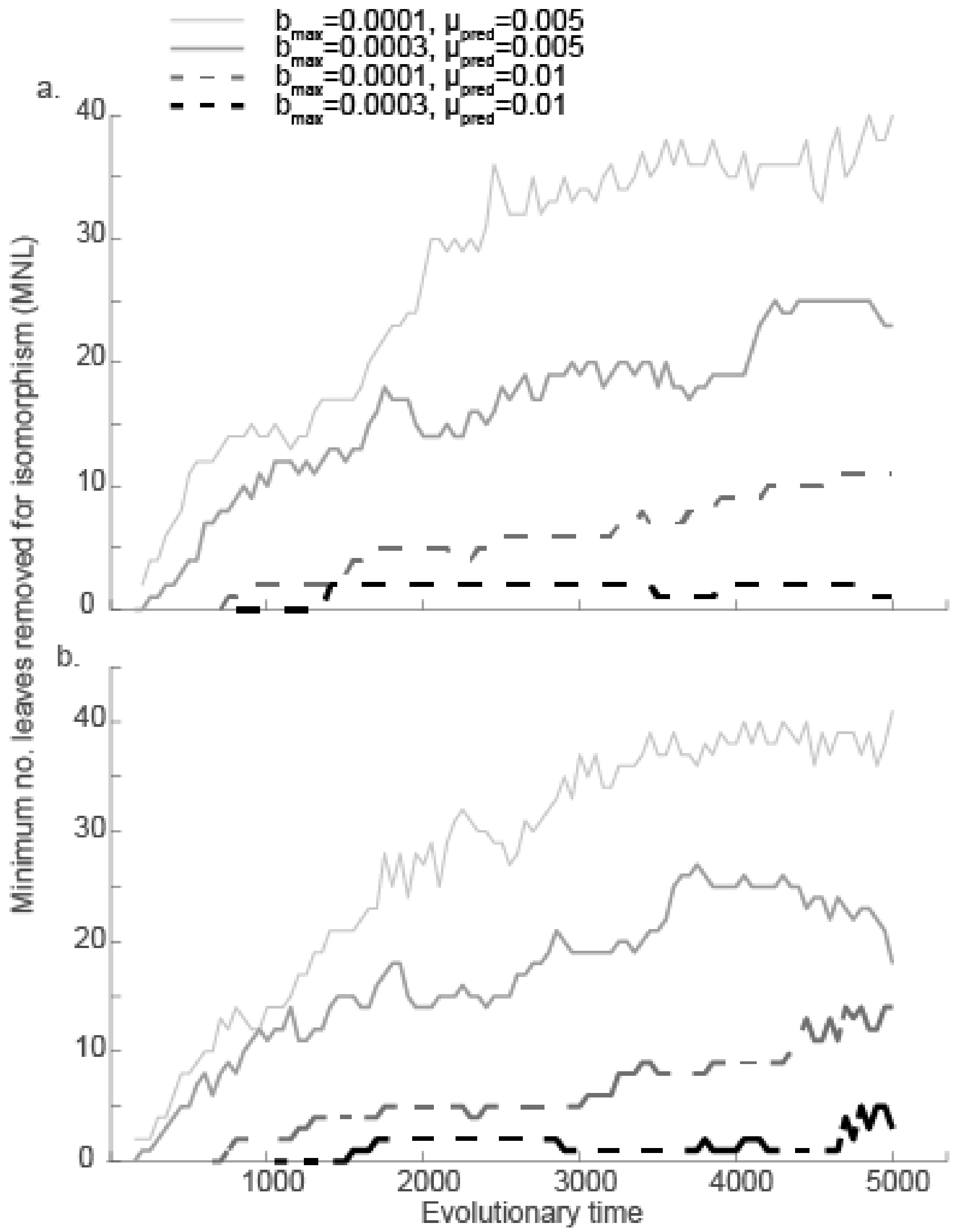

Figure S5. Congruence robustness across model replicates. Congruence for two model realizations (a, b) measured as the minimum number of leaves (in the predator and prey phylogeny) removed to reach isomorphic phylogenies, as a function of evolutionary time, for combinations of b_{max} and μ_{pred} combinations. Note, low MNL is related to high congruence.
